# Meta-analysis of proportion estimates of Extended-Spectrum-Beta-Lactamase-producing *Enterobacteriaceae* in East Africa hospitals

**DOI:** 10.1186/s13756-016-0117-4

**Published:** 2016-05-14

**Authors:** Tolbert Sonda, Happiness Kumburu, Marco van Zwetselaar, Michael Alifrangis, Ole Lund, Gibson Kibiki, Frank M. Aarestrup

**Affiliations:** Kilimanjaro Clinical Research Institute, Kilimanjaro Christian Medical Centre, Moshi, Tanzania; Kilimanjaro Christian Medical University College, Moshi, Tanzania; Centre for Medical Parasitology, Department of Immunology and Microbiology, University of Copenhagen, Copenhagen, Denmark; Department of Infectious Diseases, Copenhagen University Hospital, Copenhagen, Denmark; Centre for Genomic Epidemiology, Technical University of Denmark, Copenhagen, Denmark; Centre for Biological Sequence Analysis, Technical University of Denmark, Copenhagen, Denmark

**Keywords:** Antibiotic resistance, Extended-Spectrum-Beta-Lactamase, ESBL, *Enterobacteriaceae*, East Africa

## Abstract

**Background:**

A high proportion of Extended-Spectrum-Beta-Lactamase (ESBL) producing *Enterobacteriaceae* is causing common infections in all regions of the world. The burden of antibiotic resistance due to ESBL in East Africa is large but information is scarce and thus it is unclear how big the problem really is. To gain insight into the magnitude and molecular epidemiology of ESBL-producing *Enterobacteriaceae* in East Africa a literature search was performed in PubMed on 31 July 2015 to retrieve articles with relevant information on ESBL.

**Methods and results:**

Meta-analysis was performed to determine overall proportion estimate of ESBL-producing *Enterobacteriaceae*. A total of 4076 bacterial isolates were included in the analysis. The overall pooled proportion of ESBL-producing *Enterobacteriaceae* among included surveys done in East African hospitals was found to be 0.42 (95 % CI: 0.34–0.50). Heterogeneity (I^2^) between countries’ proportions in ESBL was significantly high (96.95 % and *p* < 0.001). The frequently detected genes encoding ESBL were CTX-M, TEM, SHV and OXA while the most infrequent reported genes were KPC and NDM.

**Conclusion:**

The available studies show a very wide variation in resistance due to ESBL between countries. This highlights a need for active surveillance systems which can help understand the actual epidemiology of ESBL, aid in formulating national or regional guidelines for proper screening of ESBL, and support developing standardized approaches for managing patients colonized with ESBL.

**Electronic supplementary material:**

The online version of this article (doi:10.1186/s13756-016-0117-4) contains supplementary material, which is available to authorized users.

## Background

The production of beta-lactamases is the most common mechanism for bacteria to acquire resistance to broad-spectrum beta-lactam antibiotics. These hydrolytic enzymes are encoded by various gene variants. TEM, named after Temoneira, is one of the first enzymes identified in Europe in the 1960s in *Escherichia coli* [1]. Since then, several other enzymes like CTX, SHV, OXA have been reported in different parts of the world [2–8]. Extended-Spectrum-Beta-Lactamase (ESBL) producing *Enterobacteriaceae* can be defined as those producing β-lactamases capable of conferring bacterial resistance to the penicillins, first-, second-, and third-generation cephalosporins, and aztreonam (but not the cephamycins or carbapenems) by hydrolysis of these antibiotics, and which are inhibited by β-lactamase inhibitors such as clavulanic acid [9].

The proportion of ESBL-producing bacteria causing common infections in all regions of the world is high, making antibiotic resistance due to ESBL being a major global public health problem [10]. Patients infected with ESBL not only have an increased risk of treatment failure, sometimes resulting in death, but also require more health-care resources. ESBL bacterial infections are becoming challenging because physicians run out of drug options. Although there might be differences in magnitude depending on region or country, ESBLs used to be considered primarily nosocomial. Currently they can be frequently found in both hospitals and communities, though magnitudes reported in community-based surveys are generally lower [4, 6–8, 11–13]. Several risk factors have been documented to be associated with ESBL acquisition, including: previous hospitalisation, previous use of antibiotics such as third generation cephalosporins, hospital overcrowding, bed sharing when hospitalised, and international travel [3, 14–19].

Due to regional differences in the ESBL proportion and distribution, therapeutic decisions should be based on local guidelines derived from local epidemiological data [20]. In resource-rich countries many surveillance systems have been set up to estimate the burden of bacterial infections including ESBL and to determine risk factors for acquisition of ESBL bacteria as well as the clinical outcomes associated with their infection [10, 21–23]. In East Africa however, only scarce and scattered information is available on ESBL epidemiology and risk factors associated with ESBL bacterial infection. When available, the data mostly originates from hospital-based studies [7, 24–28]. To gain a better insight in the proportion estimates and molecular epidemiology of ESBL-producing *Enterobacteriaceae* in East Africa, we retrieved available peer-reviewed articles and collated the information in this review article. The PRISMA statement was used to guide the meta-analysis [29]; the PRISMA checklist is available as supplemental material (Additional file 1).

## Materials and methods

### Literature search and selection

**Fig. 1:**
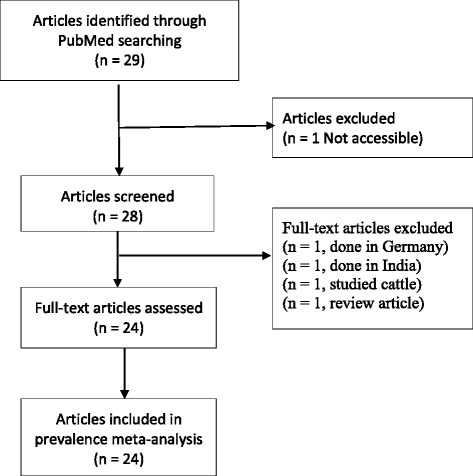
Flow diagram summarising the process of literature search and selection

We defined the East African region as the countries forming the East African Community (Burundi, Kenya, Rwanda, Tanzania and Uganda), with the addition of Ethiopia, based on the UN geographical regions definition. A PubMed search was performed on 31 July 2015 using the search query “(Burundi OR Kenya OR Rwanda OR Tanzania OR Uganda OR Ethiopia) AND (ESBL OR extended-spectrum-beta-lactamase”. A total of 29 potential articles were found. Articles studying non-human subjects, review articles, and articles describing isolates from outside the six countries mentioned above were excluded. A total of 24 articles were included (Fig. 1).

### Data extraction and analysis

For data consistency, two people extracted data independently from each article. Whenever there was discordance in the data extracted, consensus was reached by double-checking the article. Data extracted included: name of first authors, year of study (or publication if year of study was not documented), department, target population, isolate source and common species isolated. Other data extracted were the methods used to test for ESBL, the number of *Enterobacteriaceae* analysed, the number of *Enterobacteriaceae* positive for ESBL, the gene variants encoding for ESBL, and risk factors for ESBL bacterial infection.

Stata version 13.1 (College Station, Texas 77845 USA) was used to perform meta-analysis of the proportion of ESBL-producing *Enterobacteriaceae* as described by Nyaga et al. [30]. In the analysis, a random-effects meta-analysis model was used to calculate the pooled (weighted) proportion of ESBL and the I^2^ statistic (measure of inconsistency). The I^2^ statistic expresses the percentage of total variation across studies due to heterogeneity. A value of 0 % shows no observed heterogeneity, increasing values indicate increasing heterogeneity.

## Results

### Distribution of articles describing ESBL in East Africa

The 24 articles reviewed were from cross-sectional hospital-based studies. 4 (16.7 %) were from Ethiopia, 4 (16.7 %) were from Kenya, 12 (50 %) were from Tanzania, 3 (12.5 %) were from Uganda, and 1 (4.2 %) was from Rwanda. No articles were found for Burundi. 18 (75 %) of reviewed articles included patients attending in-patient and out-patient departments, 4 (16.7 %) included patients attending in-patient departments only, and 2 (8.3 %) included patients attending out-patient departments only. 20 (83.3 %) of the reviewed articles studied E. coli plus other species while 19 (79.2 %) studied K. pneumoniae plus other species.

One (4.2 %) article (from Rwanda) investigated the risk factors for infection with ESBL-producing *Enterobacteriaceae*. In this article, previous use of cephalosporins, ciprofloxacin and hospitalisation were found to be significant risk factors for ESBL bacterial infection.

### Laboratory methods used to estimate the proportion of ESBL

Sixteen (66.7 %) of the articles reviewed used Double Disk Synergy Test (DDST) alone; 2 (8.3 %) articles used PCR-sequencing alone while 6 (25 %) articles used both DDST and PCR-sequencing methods to estimate ESBL proportions.

### Proportion estimates of ESBL in East Africa

**Fig. 2:**
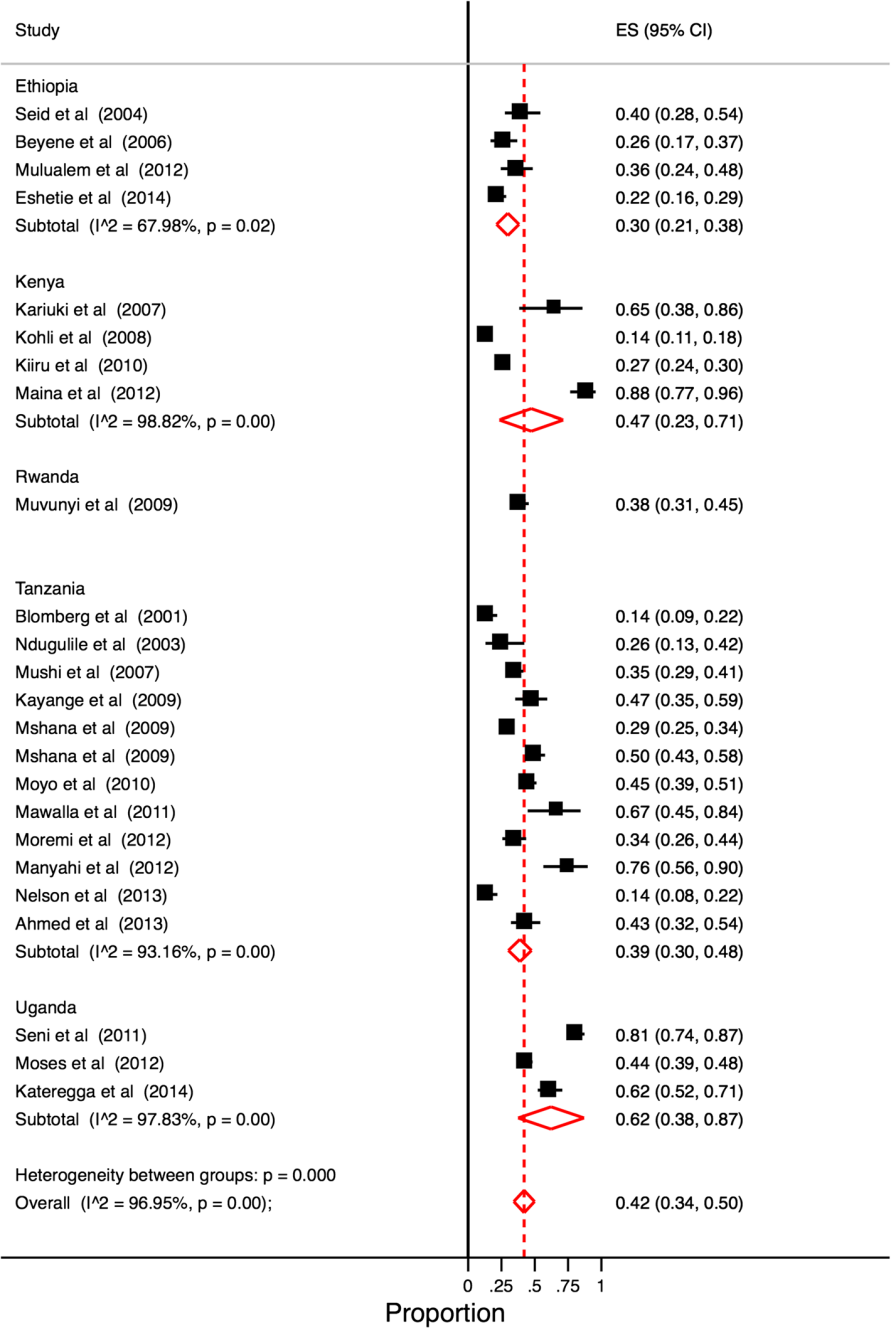
Proportion estimates of ESBL-producing Enterobacteriaceae in East African hospitals. Midpoint of each horizontal line segment shows the proportion estimate of ESBL in each study. Rhombic mark shows the pooled proportion from all studies included

Based on the available data (Fig. 2), East Africa’s overall pooled proportion of ESBL-producing *Enterobacteriaceae* was 0.42 (95 % CI: 0.34–0.50). Overall heterogeneity was significant (I^2^ 95.95 %, *p* < 0.001). The pooled proportion of ESBL-producing *Enterobacteriaceae* in Ethiopia was 0.30 (95 % CI: 0.21–0.38), I^2^ was 67.98 % and *p* = 0.02. The pooled proportion of ESBL-producing *Enterobacteriaceae* in Kenya was 0.47 (95 % CI: 0.23–0.71); I^2^ was 98.82 % and *p* < 0.001. The pooled proportion of ESBL-producing *Enterobacteriaceae* in Tanzania was 0.39 (95 % CI: 0.30–0.48); I^2^ was 93.16 % and *p* < 0.001. The pooled proportion of ESBL-producing *Enterobacteriaceae* in Uganda was 0.62 (95 % CI: 0.38–0.87); I^2^ was 97.83 % and *p* < 0.001.

**Fig. 3:**
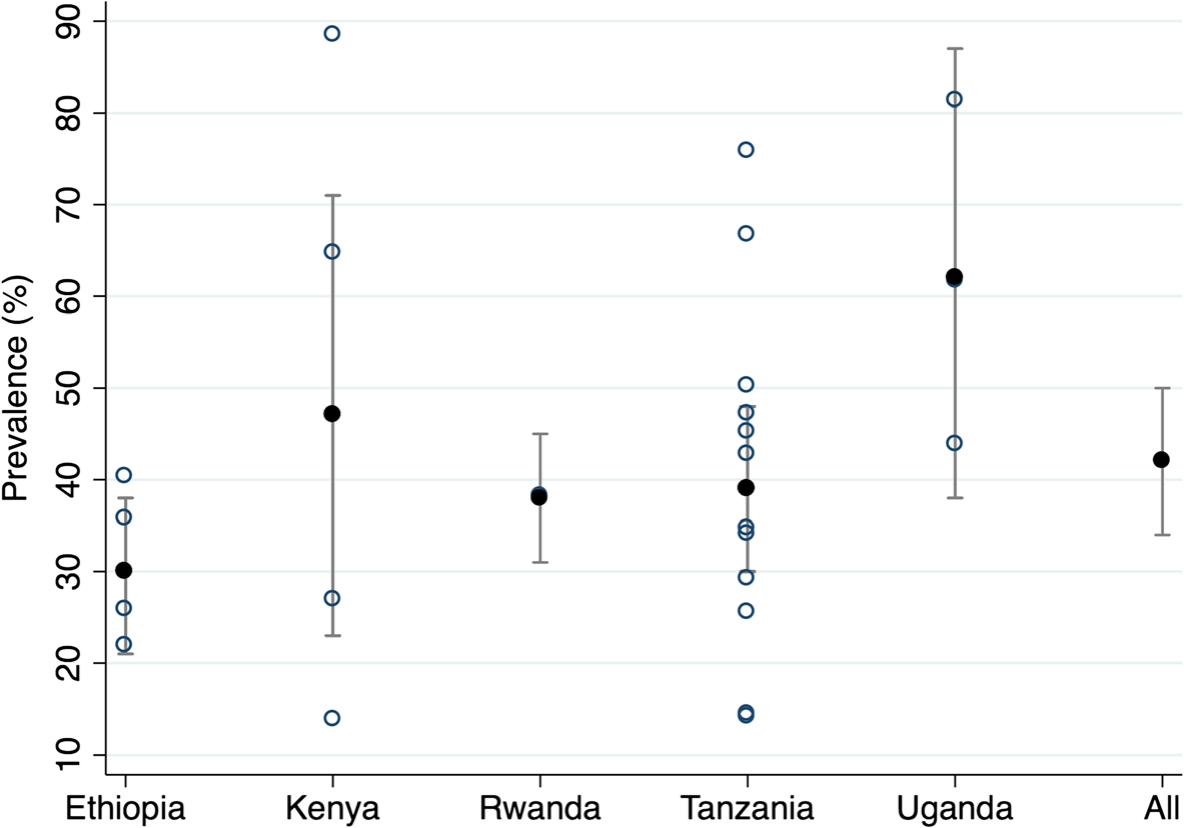
Proportion estimates of ESBL-producing Enterobacteriaceae East African hospitals with 95 % Confidence Intervals by country. Open circles show the proportion estimate of ESBL in each study. Solid circles show the pooled proportion from all studies included

The intra-country heterogeneity is reflected in the range of the estimates of the ESBL proportion. In Tanzania, the median (range) ESBL proportion estimate was 38.8 % (14.2–75.9). In Kenya the median ESBL proportion was 45.8 % (13.1–88.3). Ethiopia recorded median ESBL proportion of 30.9 % (21.9–40.4). In Uganda the recorded median ESBL proportion was 61.7 % (43.8–81.4). The single article for Rwanda reported a 38.3 % ESBL proportion (Fig. 3).

### Molecular epidemiology of ESBL in East Africa

Out of 24 reviewed articles, 8 (33.3 %) of which, 4 (50 %) from Tanzania, 3 (37.5 %) from Kenya and 1 (12.5 %) from Uganda had data on ESBL-encoding genes. The predominant ESBL-encoding genes reported were CTX-M, TEM and SHV. Five articles provided proportion estimates for these genes (Table 1). One article reported no quantitative data but identified the same three genes [12]. One article identified CTX-M-15, CMY-2 and AmpC [31], One article reported the multi resistant and rarer genes VIM (12.3 %), OXA-48 (4.9 %), KPC (3.5 %) and NDM (3.1 %) genes [42].

**Table 1:** Distribution of articles reviewed on resistance due to ESBL in East Africa hospitals and common gene variants encoding for ESBL

Country	Year	Hospital	Department	Population	Specimen	Method	Species	Isolates (*N*)	ESBL (%)	ESBL-Genes
Ethiopia [48]	2004	Regional	^a^Both	Children, adults	Urine, pus, sputum	^b^DDST	K. pneumoniae	57	42	^c^ND
Ethiopia [49]	2014	University	Both	Children, adults	Urine	DDST	K. pneumoniae, E. coli	183	22	ND
Ethiopia [50]	2006	University	Out-patient	Children	Blood, stool	DDST	Salmonella spp	81	27	ND
Ethiopia [51]	2012	University	Both	Children, adults	Urine, pus, wound, sputum, stool	DDST	E. coli	67	36	ND
Kenya [38]	2010	Not Stated	Both	Children, adults	Urine, stool, blood	DDST, PCR-Sequencing	E. coli	912	27	CTX-M (78 %), SHV (3–5 %), TEM (16 %)
Kenya [30]	2007	University	Both	Children, adults	Urine	PCR-Sequencing	E. coli	17	71	CTX-M-15 type ESBLs and CMY-2 AmpC
Kenya [52]	2008	University	Both	Children, adults	Blood	DDST	K. pneumoniae, E. coli	359	14	ND
Kenya [37]	2012	University	Both	Children, adults	Urine, pus, sputum, stool	DDST, PCR-Sequencing	K. pneumoniae, E. coli	52	89	CTX-M (88.5 %), blaSHV (25 %), TEM (34.6 %)
Rwanda [14]	2009	University	Both	Children, adults	Urine	DDST	K. pneumoniae, E. coli, others	196	38	ND
Tanzania [11]	2001	University	In-patients	Children	Blood	DDST, PCR-Sequencing	K. pneumoniae, E. coli	125	15	CTX-M, SHV, TEM
Tanzania [24]	2009	University	In-patients	Children, adults	Urine, pus, wound, blood	DDST, PCR-Sequencing	K. pneumoniae	183	50	CTX-M-15 (76 %), TEM-104 (19 %), TEM-176 (2 %), SHV-11 (3.2 %)
Tanzania [6]	2003	University	In-patients	Children, adults	Urine, wound, blood	DDST, PCR-Sequencing	E. coli, Enterobacter spp, others	39	28	TEM (55 %), SHV (64 %), CTX-M (45.4 %)
Tanzania [7]	2010	University	Both	Children, adults	Urine	DDST	K. pneumoniae, E. coli	270	45	ND
Tanzania [53]	2009	University	Both	Children, adults	Urine, pus, wound, blood	DDST	K. pneumoniae, Escherichia coli, Acinetobacter spp	377	29	ND
Tanzania [54]	2013	University	In-patients	Women, neonates	Rectal swabs	DDST	K. pneumoniae, E. coli, Enterobacter spp	113	15	ND
Tanzania [55]	2013	University	Both	Children	Urine	DDST	K. pneumoniae, E. coli, others	84	44	ND
Tanzania [56]	2012	University	Both	Children, adults	Pus	DDST	K. pneumoniae, E. coli, others	29	79	ND
Tanzania [36]	2007	University	Both	Children, adults	Urine, pus, blood	^d^PCR-Sequencing	K. pneumoniae, E. coli, P. aeruginosa, others	227	35	VIM (12.3 %), OXA-48 (4.9 %), KPC (3.5 %), NDM (3.1 %)
Tanzania [57]	2012	University	Both	Children, adults	Wound	DDST	Pseudomonas spp, Proteus spp, K. pneumoniae, E. coli	117	35	ND
Tanzania [58]	2011	University	Both	Children, adults	Wound	DDST	K. pneumoniae, E. coli, others	24	71	ND
Tanzania [59]	2009	University	Both	Children	Blood	DDST	K. pneumoniae, E. coli, others	72	49	ND
Uganda [10]	2011	Regional	Both	Children, adults	Wound	DDST	K. pneumoniae, E. coli, others	145	81	ND
Uganda [60]	2014	Regional	Both	Children, adults	Urine, blood, wound, CSF^e^	DDST	E. coli, K. pneumoniae, P. mirabilis, others	115	62	ND
Uganda [31]	2012	Regional	Both	Children, adults	Urine, pus, wound, sputum, stool, CSF, vaginal-swabs	DDST, PCR-Sequencing	K. pneumoniae, E. coli, Salmonella spp, others	484	44	CTX-M (70 %), SHV (34 %) and TEM (47 %)

^a^Both, out-and in-patients departments

^b^ *DDST* double disc synergy test

^c^ *ND* not determined

^d^ *PCR* polymerase chain reaction

^e^ *CSF* cerebrospinal fluid

## Discussion

The scarcity of studies available from the East African region warrants caution in drawing conclusions. Little information overall is available, with no studies on Burundi and only one on Rwanda. However, this review finds high proportion estimates of ESBL-producing Enterobacteriaceae across hospitals in the East African region.

The overall pooled ESBL proportion estimate for East African hospitals (42 %) is close to estimates for Ghana (49 %), Cameroon (54 %), Gabon (45 %) and Morocco (43 %) [18, 32–34]. This estimate is also close to data reported for China, where a nationwide survey that included 30 hospitals reported over 46 % resistance due to ESBL [35]. However the East African proportion is considerably higher than averages reported for resource-rich countries. For instance, the average ESBL proportion estimates reported in a nationwide hospital survey in Germany for 2012 were in the 10 to 15 % range [36]. In 2012, a study for nine US census regions reported ESBL proportion estimates in the 4 to 12 % range [37], while a 9-year survey in Japan recorded a proportion estimate of 6.3 % in 2003 increasing to 10–20 % in 2011 [38]. It should be noted that this study focused on community-acquired infections whereas the other studies concerned nosocomial-infections.

In this review, inter-country and inter-study results show a wide and statistically significant degree of variation in proportion estimates (*p* < 0.05 in all cases). There are several possible factors that may account for the variations seen in this review. The first factor is the difference in sensitivity and specificity between methods used in estimating proportions. Some studies reviewed estimated ESBL proportions using purely phenotypic methods, while others used both phenotypic and molecular-based methods. The study done in Uganda shows the proportion estimate using genotypic methods being higher (44 %) than when the same isolates were screened for ESBL using phenotypic methods (18 %) [39].

A second factor contributing to the variation in proportion estimates is type of wards or units, site of infection, type of specimen collected and whether patients were attending out-patient or in-patient departments. Hospitalised patients especially in intensive care units are generally at a higher risk of acquiring nosocomial infections, which are likely to be ESBL-producers than patients attending out-patient departments [6, 7, 40–42]. The Rwanda study [15] reports ESBL proportions of 38 % and 5.9 % among inpatients and outpatients respectively, within a single hospital. Similar findings have been documented in Cameroon where ESBL proportions were 23.1 and 6.7 % among outpatients and healthy volunteers respectively [16].

Many reports have documented the difference in ESBL proportion estimates between hospitals and congested centers (such as orphanages) versus community-based surveys [15, 16, 43–46]. The lack of any estimates for community-based ESBL carriage in East Africa underscores an urgent need for surveillance in the region. Infection control in hospitals including hand hygiene and rational antibiotic use can be effective measures to stop further spread of the ESBL-producing *Enterobacteriaceae* in both hospitals and communities.

We noted that few (33.3 %) articles investigated different genes encoding ESBL [6, 12, 24, 31, 39, 47–49]. The most common gene variants in these articles are those encoding CTX, TEM and SHV. However one study in Tanzania reported occurrence of genes such as NDM that confer resistance to Carbapenem [47]. With such little information in hand, it is difficult to devise focused and effective interventions for containing resistance. Data from other parts of the world, notably from resource-rich countries, might not be generalisable to African settings.

Risk factors associated with resistance due to ESBL are key for planning the optimal approach to managing the problem. Of the reviewed articles only the one study done in Rwanda investigated these factors. It was concluded that previous use of ciprofloxacin, third-generation cephalosporin and being an inpatient or hospitalised are risk factors for ESBL carriage [15]. Similarly, several studies done in other sub-Saharan African countries have documented the previous use of antibiotics (ciprofloxacin), previous hospitalisation, overcrowding in hospitals, and bed sharing to be associated with ESBL colonisation [14, 16, 17]. These findings are consistent with several studies done outside of Africa [50–58].

In African settings previous antibiotics use is not entirely dependent on previous hospitalisation. Over-the-counter sale of drugs or self-medication, consumption of counterfeit drugs, improper dosage and non-adherence are very common. These practices unnecessarily fuel the process of positive selection for many types of antibiotic resistance. The majority of sub-Saharan hospitals lack antibiotics resistance monitoring systems, proper antibiotic usage guidelines and proper hospital premises disinfection guidelines. If we do not put strong pragmatic measures in place our hospitals may soon turn into hotspots not only for ESBL-producing *Enterobacteriaceae,* but also for many other types of resistant microorganisms.

When interpreting the data compiled in this review, a number of limitations must be taken into account. All studies took place in university teaching or referral hospitals, which will have a different case-mix from peripheral health centres, and will generally be located in urban areas. Another source of bias is the fact that no study included community-based findings. As in any meta-analysis, the pooled proportions across studies must be interpreted with care, as protocols for observational studies are not standardised across the studies included in the review. A notable factor contributing to variation is the different mix of in- and outpatients between the studies. Finally, the authors acknowledge that more data on ESBL in East Africa may be available from sources other than those searched for this review.

## Conclusion

The burden of antibiotic resistance due to ESBL is present across East African region. Little information overall is available, and close to none for Burundi. The available studies present proportion estimates due to ESBL with a wide degree of variation. The scarcity of data on predictors, clinical outcomes, magnitudes and gene variants encoding resistance due to ESBL-producing *Enterobacteriaceae* calls for active surveillance systems, which can help understand the current epidemiology of ESBL within the region. Furthermore this can aid in developing national and regional guidelines for proper screening of ESBL as well as developing standardized approaches for managing patients colonized with ESBL-producing *Enterobacteriaceae*.

## Additional file

Additional file 1: The PRISMA checklist. (DOC 62 kb)

## References

[1] Datta N, Kontomichalou P (1965). Penicillinase synthesis controlled by infectious R factors in Enterobacteriaceae. Nature.

[2] Deng M, Zhu M-H, Li J-J, Bi S, Sheng Z-K, Hu F-S, Zhang J-J, Chen W, Xue X-W, Sheng J-F, Li L-J (2014). Molecular epidemiology and mechanisms of tigecycline resistance in clinical isolates of Acinetobacter baumannii from a Chinese university hospital. Antimicrob Agents Chemother.

[3] Feglo P, Adu-Sarkodie Y, Ayisi L, Jain R, Spurbeck RR, Springman AC, Engleberg NC, Newton DW, Xi C, Walk ST (2013). Emergence of a novel extended-spectrum-β-lactamase (ESBL)-producing, fluoroquinolone-resistant clone of extraintestinal pathogenic Escherichia coli in Kumasi, Ghana. J Clin Microbiol.

[4] Xia S, Fan X, Huang Z, Xia L, Xiao M, Chen R, Xu Y, Zhuo C (2014). Dominance of CTX-M-type extended-spectrum β-lactamase (ESBL)-producing Escherichia coli isolated from patients with community-onset and hospital-onset infection in China. PLoS One.

[5] Tängdén T, Cars O, Melhus A, Löwdin E (2010). Foreign travel is a major risk factor for colonization with Escherichia coli producing CTX-M-type extended-spectrum beta-lactamases: a prospective study with Swedish volunteers. Antimicrob Agents Chemother.

[6] Ndugulile F, Jureen R, Harthug S, Urassa W, Langeland N (2005). Extended spectrum beta-lactamases among Gram-negative bacteria of nosocomial origin from an intensive care unit of a tertiary health facility in Tanzania. BMC Infect Dis.

[7] Moyo SJ, Aboud S, Kasubi M, Lyamuya EF, Maselle SY (2010). Antimicrobial resistance among producers and non-producers of extended spectrum beta-lactamases in urinary isolates at a tertiary Hospital in Tanzania. BMC Res Notes.

[8] Mshana SE, Hain T, Domann E, Lyamuya EF, Chakraborty T, Imirzalioglu C (2013). Predominance of Klebsiella pneumoniae ST14 carrying CTX-M-15 causing neonatal sepsis in Tanzania. BMC Infect Dis.

[9] Paterson DL, Bonomo RA (2005). Extended-Spectrum -Lactamases: a clinical update. Clin Microbiol Rev.

[10] World Health Organization (2014). ANTIMICROBIAL RESISTANCE: global report on surveillance.

[11] Seni J, Najjuka CF, Kateete DP, Makobore P, Joloba ML, Kajumbula H, Kapesa A, Bwanga F (2013). Antimicrobial resistance in hospitalized surgical patients: a silently emerging public health concern in Uganda. BMC Res Notes.

[12] Blomberg B, Jureen R, Manji KP, Tamim BS, Mwakagile DSM, Urassa WK, Fataki M, Msangi V, Tellevik MG, Maselle SY, Langeland N (2005). High Rate of Fatal Cases of Pediatric Septicemia Caused by Gram-Negative Bacteria with Extended-Spectrum Beta-Lactamases in Dar es Salaam, Tanzania. J Clin Microbiol.

[13] Christopher A, Mshana SE, Kidenya BR, Hokororo A, Morona D (2013). Bacteremia and resistant gram-negative pathogens among under-fives in Tanzania. Ital J Pediatr.

[14] Lonchel CM, Melin P (2013). Extended-spectrum β -lactamase-producing Enterobacteriaceae in Cameroonian hospitals. Eur J Clin Microbiol Infect Dis.

[15] Muvunyi CM, Masaisa F, Bayingana C, Mutesa L, Musemakweri A, Muhirwa G, Claeys GW (2011). Decreased susceptibility to commonly used antimicrobial agents in bacterial pathogens isolated from urinary tract infections in Rwanda: need for new antimicrobial guidelines. AmJTrop Med Hyg.

[16] Lonchel CM, Meex C, Gangoué-Piéboji J, Boreux R, Assoumou M-CO, Melin P, De Mol P (2012). Proportion of extended-spectrum ß-lactamase-producing Enterobacteriaceae in community setting in Ngaoundere, Cameroon. BMC Infect Dis.

[17] Isendahl J, Turlej-Rogacka A, Manjuba C, Rodrigues A, Giske CG, Nauclér P (2012). Fecal carriage of ESBL-producing E. coli and K. pneumoniae in children in Guinea-Bissau: a hospital-based cross-sectional study. PLoS One.

[18] Obeng-Nkrumah N, Twum-Danso K, Krogfelt K a, Newman MJ (2013). High levels of extended-spectrum beta-lactamases in a major teaching hospital in Ghana: the need for regular monitoring and evaluation of antibiotic resistance. AmJTrop Med Hyg.

[19] Wintersdorff CJH Von, Penders J, Stobberingh EE, Lashof AMLO, Hoebe CJPA, Savelkoul PHM, Wolffs PFG. High Rates of Antimicrobial Drug Resistance Gene Acquisition after International Travel, the Netherlands. 2014;20:649-657 10.3201/eid2004.131718 PMC3966371 24655888

[20] García-Tello A, Gimbernat H, Redondo C, Arana DM, Cacho J, Angulo JC. Extended-spectrum beta-lactamases in urinary tract infections caused by Enterobacteria: Understanding and guidelines for action. Actas urologicas espanolas. 2014;38:678-684. 10.1016/j.acuro.2014.05.004 24984581

[21] Muellner P, Pleydell E, Pirie R, Baker MG, Campbell D, Carter PE, French NP. Molecular-based surveillance of campylobacteriosis in New Zealand – from source attribution to genomic epidemiology. Euro Surveill. 2013;18. Available online: http://www.eurosurveillance.org/ViewArticle.aspx?ArticleId=20365. 23351655

[22] Bathoorn E, Friedrich AW, Zhou K, Arends JP, Borst DM, Grundmann H, Rossen JW. Latent introduction to the Netherlands of multiple antibiotic resistance including NDM-1 after hospitalisation in Egypt, August 2013. Euro Surveill. 2013;18. Available online: http://www.eurosurveillance.org/ViewArticle.aspx?ArticleId=20610. 10.2807/1560-7917.es2013.18.42.20610 24176580

[23] Knetsch C, Lawley T, Hensgens M, Corver J, Wilcox M, Kuijper E. Current application and future perspectives of molecular typing methods to study Clostridium difficile infections. Euro Surveill. 2013;18. Available online: http://www.eurosurveillance.org/ViewArticle.aspx?ArticleId=20381. 10.2807/ese.18.04.20381-en 23369393

[24] Mshana SE, Hain T, Domann E, Lyamuya EF, Chakraborty T (2013). Predominance of Klebsiella pneumoniae ST14 carrying CTX-M-15 causing neonatal sepsis in Tanzania. BMC Infect Dis.

[25] Blomberg B, Manji KP, Urassa WK, Tamim BS, Mwakagile DSM, Jureen R, Msangi V, Tellevik MG, Holberg-petersen M, Harthug S, Maselle SY, Langeland N (2007). Antimicrobial resistance predicts death in Tanzanian children with bloodstream infections : a prospective cohort study. BMC Infect Dis.

[26] Mshana SE, Matee M, Rweyemamu M (2013). Antimicrobial resistance in human and animal pathogens in Zambia, Democratic Republic of Congo, Mozambique and Tanzania : an urgent need of a sustainable surveillance system. Ann Clin Microbiol Antimicrob.

[27] Meremo A, Mshana SE, Kidenya BR, Kabangila R, Peck R, Kataraihya JB (2012). High prevalence of Non – typhoid salmonella bacteraemia among febrile HIV adult patients admitted at a tertiary Hospital, North-Western Tanzania. Int Arch Med.

[28] Blomberg B, Mwakagile DSM, Urassa WK, Maselle SY, Mashurano M, Digranes A, Harthug S, Langeland N (2004). Surveillance of antimicrobial resistance at a tertiary hospital in Tanzania. BMC Public Health.

[29] Moher D, Liberati A, Tetzlaff J, Altman DG (2009). Preferred reporting items for systematic reviews and meta-analyses: the PRISMA statement. PLoS Med.

[30] Nyaga VN, Arbyn M, Aerts M (2014). Metaprop : a Stata command to perform meta-analysis of binomial data. Arch Public Health.

[31] Kariuki S, Revathi G, Corkill J, Kiiru J, Mwituria J, Mirza N, Hart CA (2007). Escherichia coli from community-acquired urinary tract infections resistant to fluoroquinolones and extended-spectrum beta-lactams. J Infect Dev Ctries.

[32] Schaumburg F, Alabi A, Kokou C, Grobusch MP, Köck R, Kaba H, Becker K, Adegnika AA, Kremsner PG, Peters G, Mellmann A (2013). High burden of extended-spectrum β-lactamase-producing Enterobacteriaceae in Gabon. J Antimicrob Chemother.

[33] Magoué CL, Melin P, Gangoué-Piéboji J, Okomo Assoumou M-C, Boreux R, De Mol P (2013). Prevalence and spread of extended-spectrum β-lactamase-producing Enterobacteriaceae in Ngaoundere, Cameroon. Clin Microbiol Infect.

[34] Girlich D, Bouihat N, Poirel L, Benouda A, Nordmann P (2014). High rate of faecal carriage of extended-spectrum β-lactamase and OXA-48 carbapenemase-producing Enterobacteriaceae at a university hospital in Morocco. Clin Microbiol Infect.

[35] Zhang J, Zheng B, Zhao L, Wei Z, Ji J, Li L, Xiao Y (2014). Nationwide high prevalence of CTX-M and an increase of CTX-M-55 in Escherichia coli isolated from patients with community-onset infections in Chinese county hospitals. BMC Infect Dis.

[36] Leistner R, Schröder C, Geffers C, Breier A-C, Gastmeier P, Behnke M (2015). Regional distribution of nosocomial infections due to ESBL-positive Enterobacteriaceae in Germany: data from the German National Reference Center for the Surveillance of Nosocomial Infections (KISS). Clin Microbiol Infect.

[37] Castanheira M, Farrell SE, Krause KM, Jones RN, Sader HS (2014). Contemporary diversity of β-lactamases among Enterobacteriaceae in the nine U.S. census regions and ceftazidime-avibactam activity tested against isolates producing the most prevalent β-lactamase groups. Antimicrob Agents Chemother.

[38] Chong Y, Shimoda S, Yakushiji H, Ito Y, Miyamoto T, Kamimura T, Shimono N, Akashi K (2013). Community spread of extended-spectrum β-lactamase-producing Escherichia coli, Klebsiella pneumoniae and Proteus mirabilis: a long-term study in Japan. J Med Microbiol.

[39] Moses A, Bwanga F, Boum Y, Bazira J (2014). Prevalence and Genotypic Characterization of Extended-Spectrum Beta-Lactamases Produced by Gram Negative Bacilli at a Tertiary Care Hospital in Rural South Western Uganda. Br Microbiology Res J.

[40] Manyahi J, Matee MI, Majigo M, Moyo S, Mshana SE, Lyamuya EF (2014). Predominance of multi-drug resistant bacterial pathogens causing surgical site infections in Muhimbili National Hospital, Tanzania. BMC Res Notes.

[41] Mshana SE, Imirzalioglu C, Hain T, Domann E, Lyamuya EF, Chakraborty T (2011). Multiple ST clonal complexes, with a predominance of ST131, of Escherichia coli harbouring blaCTX-M-15 in a tertiary hospital in Tanzania. Clin Microbiol Infect.

[42] Nelson E, Kayega J, Seni J, Mushi MF, Kidenya BR, Hokororo A, Zuechner A, Kihunrwa A, Mshana SE (2014). Evaluation of existence and transmission of extended spectrum beta lactamase producing bacteria from post-delivery women to neonates at Bugando Medical Center, Mwanza-Tanzania. BMC Res Notes.

[43] Suh Yah C (2010). Plasmid-encoded multidrug resistance: a case study of Salmonella and Shigella from enteric diarrhea sources among humans. Biol Res.

[44] Sire J-M, Nabeth P, Perrier-Gros-Claude J-D, Bahsoun I, Siby T, Macondo EA, Gaye-Diallo A, Guyomard S, Seck A, Breurec S, Garin B (2007). Antimicrobial resistance in outpatient Escherichia coli urinary isolates in Dakar, Senegal. J Infect Dev Ctries.

[45] Bourjilat F, Bouchrif B, Dersi N, Claude JDPG, Amarouch H, Timinouni M (2011). Emergence of extended-spectrum beta-lactamases-producing Escherichia coli in community-acquired urinary infections in Casablanca, Morocco. J Infect Dev Ctries.

[46] Frank T, Arlet G, Gautier V, Talarmin A, Bercion R (2006). Extended-spectrum beta-lactamase-producing Enterobacteriaceae, Central African Republic. Emerg Infect Dis.

[47] Mushi MF, Mshana SE, Imirzalioglu C, Bwanga F (2014). Carbapenemase Genes among Multidrug Resistant Gram Negative Clinical Isolates from a Tertiary Hospital in Mwanza, Tanzania. BioMed Res Int.

[48] Maina D, Revathi G, Kariuki S, Ozwara H (2012). Genotypes and cephalosporin susceptibility in extended-spectrum beta-lactamase producing enterobacteriaceae in the community. J Infect Dev Ctries.

[49] Kiiru J, Kariuki S, Goddeeris BM, Butaye P (2012). Analysis of β-lactamase phenotypes and carriage of selected β-lactamase genes among Escherichia coli strains obtained from Kenyan patients during an 18-year period. BMC Microbiol.

[50] Gudiol C, Calatayud L, Garcia-Vidal C, Lora-Tamayo J, Cisnal M, Duarte R, Arnan M, Marin M, Carratalà J, Gudiol F (2010). Bacteraemia due to extended-spectrum beta-lactamase-producing Escherichia coli (ESBL-EC) in cancer patients: clinical features, risk factors, molecular epidemiology and outcome. J Antimicrob Chemother.

[51] Søraas A, Sundsfjord A, Sandven I, Brunborg C, Jenum P a (2013). Risk factors for community-acquired urinary tract infections caused by ESBL-producing enterobacteriaceae--a case–control study in a low prevalence country. PLoS One.

[52] Superti SV, Augusti G, Zavascki AP (2009). Risk factors for and mortality of extended-spectrum-β-lactamase-producing Klebsiella pneumoniae and Escherichia coli nosocomial bloodstream infections. Rev Inst Med Trop Sao Paulo.

[53] Cornejo-Juárez P, Pérez-Jiménez C, Silva-Sánchez J, Velázquez-Acosta C, González-Lara F, Reyna-Flores F, Sánchez-Pérez A, Volkow-Fernández P (2012). Molecular analysis and risk factors for Escherichia coli producing extended-spectrum β-lactamase bloodstream infection in hematological malignancies. PLoS One.

[54] Kaya O, Akcam FZ, Gonen I, Unal O, Ceylan T (2013). Original Article Risk factors for bacteremia due to extended-spectrum beta-lactamase- producing Escherichia coli in a Turkish hospital. J Infect Dev Ctries.

[55] Han JH, Kasahara K, Edelstein PH, Bilker WB, Lautenbach E, Presbyterian P (2012). Risk Factors for Infection or Colonization with CTX-M Extended-. Antimicrob Agents Chemother.

[56] Demirdag K, Hosoglu S (2010). Epidemiology and risk factors for ESBL-producing Klebsiella pneumoniae : a case control study. J Infect Dev Ctries.

[57] Silva N, Oliveira M, Bandeira AC, Brites C (2006). Risk factors for infection by extended-spectrum beta-lactamase producing Klebsiella pneumoniae in a tertiary hospital in Salvador, Brazil. Braz J Infect Dis.

[58] Tumbarello M, Spanu T, Sanguinetti M, Citton R, Montuori E, Leone F, Fadda G, Cauda R (2006). Bloodstream Infections Caused by Risk Factors, Molecular Epidemiology, and Clinical Outcome. Antimicrob Agents Chemother.

[59] Kayange N, Kamugisha E, Mwizamholya DL, Jeremiah S, Mshana SE: Predictors of positive blood culture and deaths among neonates with suspected neonatal sepsis in a tertiary hospital, Mwanza-Tanzania. BMC pediatrics 2010;10:1–9. 10.1186/1471-2431-10-39 PMC2889942 20525358

[60] Kateregga JN, Kantume R, Atuhaire C, Lubowa MN, Ndukui JG: Phenotypic expression and prevalence of ESBL-producing Enterobacteriaceae in samples collected from patients in various wards of Mulago Hospital, Uganda. BMC pharmacology & toxicology 2015;16:1–6. 10.1186/s40360-015-0013-1 PMC4451872 26031914

